# Broad respiratory testing to identify SARS-CoV-2 viral co-circulation and inform diagnostic stewardship in the COVID-19 pandemic

**DOI:** 10.1186/s12985-021-01545-9

**Published:** 2021-05-01

**Authors:** Natalie C. Marshall, Ruwandi M. Kariyawasam, Nathan Zelyas, Jamil N. Kanji, Mathew A. Diggle

**Affiliations:** 1 Division of Diagnostic and Applied Microbiology, Department of Laboratory Medicine and Pathology, Faculty of Medicine and Dentistry, University of Alberta, 2B4.01, Walter C. Mackenzie Centre, Provincial Laboratory of Public Health, 8440 - 112 Street, Edmonton, AB T6G 2J2 Canada; 2 Alberta Precision Laboratories – Public Health Laboratory (ProvLab), Edmonton, AB Canada; 3 Division of Infectious Diseases, Faculty of Medicine & Dentistry, University of Alberta, Edmonton, AB Canada

**Keywords:** COVID-19, SARS-CoV-2, Endemic coronaviruses, Coronavirus 229E, human, Coronavirus NL63, human, Coronavirus OC43, human, Respiratory viruses, Multiplex testing, Diagnostic stewardship

## Abstract

**Background:**

SARS-CoV-2 infection can present with a broad clinical differential that includes many other respiratory viruses; therefore, accurate tests are crucial to distinguish true COVID-19 cases from pathogens that do not require urgent public health interventions. Co-circulation of other respiratory viruses is largely unknown during the COVID-19 pandemic but would inform strategies to rapidly and accurately test patients with respiratory symptoms.

**Methods:**

This study retrospectively examined 298,415 respiratory specimens collected from symptomatic patients for SARS-CoV-2 testing in the three months since COVID-19 was initially documented in the province of Alberta, Canada (March-May, 2020). By focusing on 52,285 specimens that were also tested with the Luminex *Respiratory Pathogen Panel* for 17 other pathogens, this study examines the prevalence of 18 potentially co-circulating pathogens and their relative rates in prior years versus since COVID-19 emerged, including four endemic coronaviruses.

**Results:**

SARS-CoV-2 was identified in 2.2% of all specimens. Parallel broad multiplex testing detected additional pathogens in only 3.4% of these SARS-CoV-2-positive specimens: significantly less than in SARS-CoV-2-negative specimens (*p* < 0.0001), suggesting very low rates of SARS-CoV-2 co-infection. Furthermore, the overall co-infection rate was significantly lower among specimens with SARS-CoV-2 detected (*p* < 0.0001). Finally, less than 0.005% of all specimens tested positive for both SARS-CoV-2 and any of the four endemic coronaviruses tested, strongly suggesting neither co-infection nor cross-reactivity between these coronaviruses.

**Conclusions:**

Broad respiratory pathogen testing rarely detected additional pathogens in SARS-CoV-2-positive specimens. While helpful to understand co-circulation of respiratory viruses causing similar symptoms as COVID-19, ultimately these broad tests were resource-intensive and inflexible in a time when clinical laboratories face unprecedented demand for respiratory virus testing, with further increases expected during influenza season. A transition from broad, multiplex tests toward streamlined diagnostic algorithms targeting respiratory pathogens of public health concern could simultaneously reduce the overall burden on clinical laboratories while prioritizing testing of pathogens of public health importance. This is particularly valuable with ongoing strains on testing resources, exacerbated during influenza seasons.

## Background

Severe acute respiratory syndrome coronavirus 2 (SARS-CoV-2) has caused significant disease (COVID-19) and deaths worldwide [1]. COVID-19 symptoms range from mild respiratory illness to severe pneumonia, encompassing a wide clinical differential of numerous respiratory pathogens. Therefore, when patients present with COVID-19-like symptoms, highly accurate laboratory tests are essential to distinguish true COVID-19 cases and initiate public health steps to limit further SARS-CoV-2 spread.

Since SARS-CoV-2 emerged, there has been little research on the concurrent circulation of these other respiratory viruses, which had been the subject of broad surveillance in the years prior [2–5]: only a handful of case reports have described co-infections between SARS-CoV-2 and other respiratory viruses able to cause similar symptoms [6–13]. With unprecedented demand on clinical diagnostics, re-prioritized surveillance is one of many ways that laboratories have had to prioritize and adapt throughout this pandemic [14–17]. However, understanding current virus co-circulation could help to develop evidence-based strategies to better tackle the pandemic in future pandemic surges and the approaching influenza season, as exponentially increasing numbers of patients require testing for COVID-19-like symptoms.

Furthermore, co-circulation of SARS-CoV-2 and endemic coronaviruses (eCoVs) may pose a particular diagnostic challenge due to potential cross-reactivity of SARS-CoV-2 with pre-existing assays targeting eCoVs and vice versa. While laboratories must perform in-lab validations of the specificity of their assays, in situ studies are an essential complement, showing real-world data that may indicate necessary and opportune changes in diagnostic approaches.

Accordingly, we sought to conduct a retrospective analysis of all specimens submitted to the provincial public health laboratory in Alberta, Canada for SARS-CoV-2 simultaneously tested for 17 additional respiratory pathogens—including influenza viruses and endemic coronaviruses—to inform our diagnostic algorithms during the COVID-19 pandemic and assess for potential co-infection or cross-reactivity of SARS-CoV-2 and eCoVs in a clinical setting.

## Methods

### Multiplex respiratory testing

The NxTAG Respiratory Pathogen Panel (RPP; Luminex) was used to test respiratory specimens from symptomatic patients admitted to hospital, emergency departments, in long-term care, or amid a suspected respiratory outbreak in the community. Following collection, respiratory specimens were transported to the provincial Public Health Laboratories (ProvLab) for testing. The RPP was validated to detect nucleic acids from 17 respiratory pathogens: influenza viruses A-B, parainfluenza viruses 1–4, respiratory syncytial viruses A-B, rhinovirus/enterovirus, adenovirus, human metapneumovirus, four eCoVs (229E, NL63, OC43, HKU1), and *Mycoplasma pneumoniae*.

### SARS-CoV-2 testing

SARS-CoV-2 testing was performed at ProvLab using multiple assays with different analytical sensitivities (Sn): singleplex and multiplex laboratory-developed tests using the TaqMan Fast Virus One-Step real-time reverse transcription (RT)-PCR Master Mix (ABI; Sn = 145 and 375 copies/mL, respectively [18]), or the Roche cobas 6800 SARS-CoV-2 test (Sn = 25 copies/mL [19]). The Seegene Allplex 2019-nCoV assay was also used outside of ProvLab (Sn = 100 copies/reaction [20]) and two rapid tests were employed across the province: DiaSorin Simplexa COVID-19 Direct (Sn = 242 copies/mL [21]) and Cepheid GeneXpert Xpress SARS-CoV-2 (Sn = 250 copies/mL [22]). No assays reported cross-reactivity with other respiratory pathogens, with the exceptions of three tests that cross-reacted with SARS-CoV-1: the cobas, the multiplex RT-PCR assay, and the GeneXpert, as previously reported [18].

### Data collection and exclusion

SARS-CoV-2 and RPP tests performed between January 1–June 6, 2020, and RPPs between January 1–June 6 in 2018/2019 were compiled. Only tests from validated, respiratory tract specimen types and from patients with a primary address in Alberta were assessed. Analyses of coronavirus test positivity in 2020 focused on March 7–May 28, wherein RPP testing criteria were consistent with those used in prior years, except for providing RPP tests to symptomatic patients in long-term care in 2020.

### Statistics

Differences between categorical variables were compared using Fisher’s exact test or Chi-square analysis. Continuous variables were compared by Kruskal–Wallis test or Student’s t-test.

## Results

### Respiratory virus testing in Alberta during the COVID-19 pandemic

**Fig. 1:**
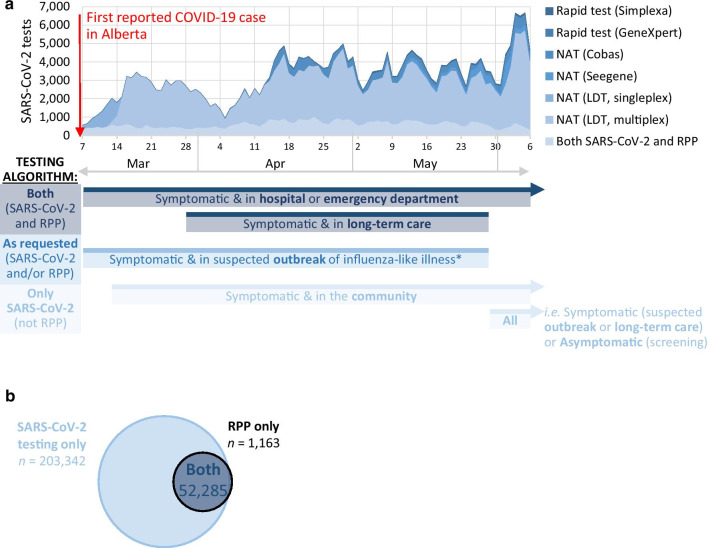
Respiratory specimen collection and testing following the first routinely reported COVID-19 case in Alberta, Canada. **a** Respiratory specimens collected for SARS-CoV-2 testing in Alberta and corresponding testing criteria. **b** Respiratory specimens tested between March 7–May 28, 2020. *NAT* nucleic acid test, *RPP* Respiratory Pathogen Panel, *LDT* Laboratory-developed test

On March 5, 2020, Alberta reported its first identified case of COVID-19^1^; this triggered a change in province-wide testing algorithms (Fig. 1a). In the following three months, 298,415 respiratory specimens were collected and sent to the Alberta provincial laboratory for SARS-CoV-2 nucleic acid testing. During this time, clinical and public health criteria (as outlined in Fig. 1a) were established to identify specimens to test in parallel for other respiratory pathogens using the multiplex, syndromic Respiratory Pathogen Panel (RPP) test. Notably, the RPP testing criteria used between March 7–May 28, 2020 were largely consistent with those used in previous years; therefore, this study focused on specimens collected March 7–May 28, 2020 to examine coronaviruses and other respiratory viruses during COVID-19 emergence in Alberta (Fig. 1a). Between March 7–May 28, 2020, 255,627 respiratory specimens were collected for SARS-CoV-2 testing, of which 52,285 were also tested by RPP for 17 other respiratory pathogens (Fig. 1b), identifying 6,717 COVID-19 cases, including 1,020 patients from whom 1,141 specimens were SARS-CoV-2-positive and also tested by RPP. In this same period, 97.4% of the respiratory specimens tested by RPP also received a SARS-CoV-2 test (*n* = 52,285/53,661; Fig. 1b).

### Broad, syndromic testing of SARS-CoV-2-positive specimens rarely identified other pathogens

To assess co-circulation of (or cross-reactivity with) other respiratory pathogens causing COVID-19-like symptoms in a clinical setting, we further examined the 1,141 specimens from which SARS-CoV-2 was detected. Of these, 96.6% were negative for all 17 RPP pathogens (Table 1). The most commonly co-identified targets were entero/rhinovirus and adenovirus (2.3 and 0.7% of specimens, respectively). An eCoV was detected in only 0.2% of all SARS-CoV-2-positive specimens, with NL63 detected most commonly among the eCoVs (*n* = 2; Table 2). In total, parallel RPP testing identified additional pathogens in 3.4% of SARS-CoV-2-positive specimens.

**Table 1:** Prevalence of RPP target respiratory pathogens among SARS-CoV-2-positive and -negative specimens

RPP targets detected	SARS-CoV-2 detected (*n* = 1 141)		SARS-CoV-2 not detected (*n* = 51 129)	
	%	#	%	#
0	96.6	1102	88.7	45,359
1	3.3	38	10.6	5408
2	0.1	1	0.7	340
3	-	0	< 0.1	17
4	-	0	< 0.1	5

*RPP* Respiratory Pathogen Panel, *NAT* nucleic acid test. Fifteen specimens are not shown: 13 where patient demographics could not be verified, one due to an error in both the SARS-CoV-2 NAT and the RPP, and one where the SARS-CoV-2 test internal control failed. Of these 15, 14 were negative for all RPP targets, and one resulted in an error for both the RPP and SARS-CoV-2 tests

**Table 2:** Prevalence of RPP target pathogens among SARS-CoV-2-positive and -negative specimens

The RPP target detected	Specimens with SARS-CoV-2 detected				Specimens with SARS-CoV-2 not detected					
	Overall (*n* = 1 141)		≥ 1 RPP target also detected (*n* = 39)		Overall (*n* = 51,129)		1 RPP target detected (*n* = 5 322)		≥ 2 RPP target detected (*n* = 362)	
	**%**	#	**%**	#	**%**	#	**%**	#	**%**	#
**Endemic coronaviruses**										
229E	**–**	0	**–**	0	**0.2**	98	**1.7**	89	**2.5**	9
NL63	**0.2**	2	**5.1**	2	**1.1**	565	**9.2**	488	**19.6**	71
OC43	**–**	0	**–**	0	**0.3**	140	**2.4**	128	**2.7**	10
HKU1	**–**	0	**–**	0	**0.3**	136	**2.2**	119	**4.7**	17
**Influenza viruses**										
A	**0.1**	1	**2.6**	1	**1.1**	555	**9.4**	502	**12.2**	44
B	**–**	0	**–**	0	**0.5**	236	**3.9**	207	**7.5**	27
**Respiratory syncytial virus**										
A	**–**	0	**–**	0	**0.7**	379	**6.0**	319	**16.0**	58
B	**–**	0	**–**	0	**0.1**	59	**1.0**	55	**1.1**	4
**Parainfluenza viruses**										
1	**–**	0	**–**	0	**0.1**	45	**0.6**	33	**2.8**	10
2	**0.1**	1	**2.6**	1	**0.1**	43	**0.6**	33	**2.8**	10
3	**–**	0	**–**	0	**0.1**	56	**0.9**	49	**1.7**	6
4	**–**	0	**–**	0	**0.2**	89	**1.2**	62	**7.2**	26
**Enterovirus/rhinovirus**	**2.3**	26	**66.7**	26	**4.3**	2 201	**36.2**	1 925	**64.9**	235
**Human metapneumovirus**	**0.1**	1	**2.6**	1	**1.6**	820	**13.6**	725	**24.0**	87
**Adenovirus**	**0.7**	8	**20.5**	8	**0.9**	441	**6.1**	324	**29.3**	106
***M. pneumoniae***	**0.1**	1	**2.6**	1	**0.6**	296	**5.0**	264	**8.6**	31

Bold to just highlight all of the data in the percentage columns (every other column)

*RPP* Respiratory Pathogen Panel

### SARS-CoV-2-negative specimens were more likely to contain multiple respiratory pathogens by broad testing

In specimens where SARS-CoV-2 was not detected, 88.7% were also negative for all 17 RPP targets, 10.6% had one target identified, and 0.7% had multiple RPP targets identified (Table 1). The most targets identified in a single specimen was four (Table 1). Enterovirus/rhinovirus remained the most commonly identified target from co-infections (4.3%), though human metapneumovirus was detected next most commonly (1.6%; Table 2). eCoVs were detected in 1.8% of SARS-CoV-2-negative specimens (*p* < 0.0001) and the predominant eCoV detected in Alberta in 2020 was NL63, accounting for 100.0% and 60.5% of the eCoV-positive specimens among SARS-CoV-2-positive and -negative specimens, respectively (*n* = 2/2 and 565/933). In total, 401 specimens had more than one respiratory pathogen detected in this study, including 39 (3.4%) of the SARS-CoV-2-positive specimens and 362 (0.7%) of the SARS-CoV-2-negative specimens (Table 1). Overall, respiratory specimens that tested negative for SARS-CoV-2 were more likely to have an RPP target detected and more likely to have multiple RPP targets identified from the same specimen (*p* < 0.0001; Table 2).

### Endemic coronavirus positivity rates were lower during the COVID-19 pandemic compared with prior years

**Fig. 2:**
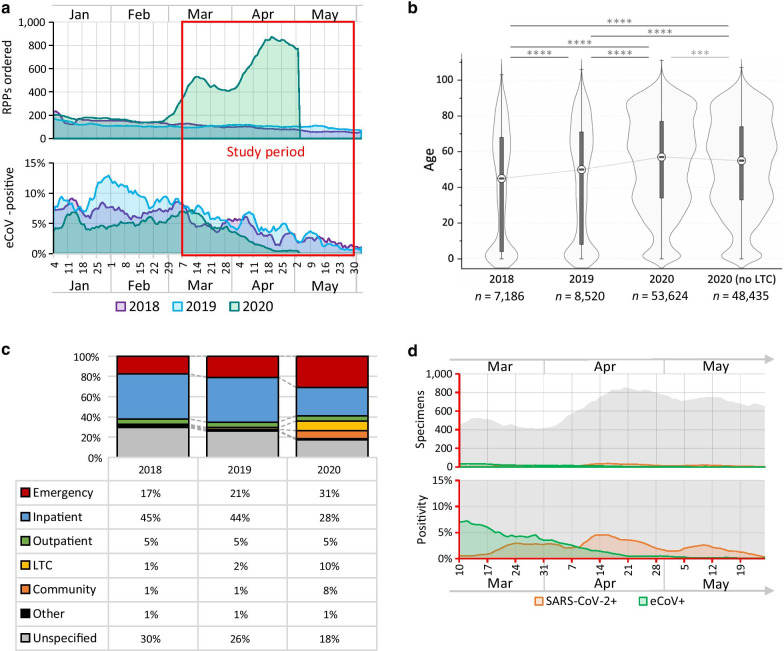
Trends in respiratory testing and positivity from 2018–2020 across Alberta. **a** A seven-day moving average of RPP testing and eCoV positivity from respiratory specimens collected between Jan 1–Jun 6 of each year. **b** Violin plot of patient age for all RPP specimens collected between Jan 1–Jun 6 of each year. Specimens were excluded if age was unknown. **c** Patient category at the time of RPP test collection over the study period (Mar 7–May 28, 2020). ‘Other’ locations included correctional facilities, armed forces, and Medical Examiner specimens. **d** A seven-day moving average of respiratory specimens tested by both RPP and SARS-CoV-2 assays in the study period (Mar 7–May 28, 2020) and respective positivity. ***p < 0.001. ****p < 0.0001. White circle, median. Whiskers, range within 1.5 × interquartile range. Dark grey box, 25th–75th percentile. *LTC* long-term care

eCoV incidence in 2020 was compared with previous years to identify any increase in positivity potentially caused by SARS-CoV-2 cross-reactivity. Between March 7 and May 28 of 2018–2020, 69,378 respiratory specimens were tested by RPP. From this period in 2020 alone, 53,661 respiratory specimens were tested by RPP, and 97.4% of these also received a SARS-CoV-2 NAT (*n* = 52,285/53,661; Table 3; Fig. 1b). A significantly smaller proportion of specimens were eCoV-positive in 2020 compared with previous years (*p* < 0.0001) and positivity decreased more sharply over time than in prior years (Fig. 2a).

**Table 3:** Demographic data of patients with RPP tests between March 7–May 28 from 2018, 2019, and 2020

Year	RPP tests	Sex, male	Age, years			eCoV detected, % (#)				
			Median (range)	< 5	> 60	299E	NL63	OC43	HKU1	Multiple
2018	7197	50.5%	45.0 (0–103)	25.8%	35.1%	0.5% (34)	2.6% (185)	0.8% (60)	0.2% (12)	< 0.1% (1)
2019	8520	49.5%	50.0 (0–106)	20.8%	39.9%	2.0% (172)	1.3% (112)	1.1% (92)	0.1% (7)	< 0.1% (4)
2020	53,661	47.0%	57.0 (0–111)	5.1%	46.0%	0.2% (99)	1.1% (578)	0.3% (145)	0.3% (136)	< 0.1% (6)
Significance		****^a^	****^b^	****^c^		****^c^				

*RPP* respiratory pathogen panel

****p < 0.0001

^a^Fisher’s exact test

^b^Kruskal–Wallis test

^c^Chi-square test for trend

### Demographics of patients receiving multiplex respiratory testing changed significantly between 2020 and prior years

Importantly, the demographics of RPP-tested patients in 2020 differed significantly from prior years, with fewer male patients, fewer children under 5 years old, higher overall median patient age, and greater proportion over 60 years old (*p* < 0.0001 for all; Table 3). Although RPP demand surged in 2020, testing criteria did not change between 2018–2020 except to include symptomatic long-term care patients. While long-term care testing did significantly increase the age of tested patients in 2020, overall (*p* < 0.001), the patient age increased significantly from previous years even when these patients were excluded (*p* < 0.0001; Fig. 2b,c), suggesting substantial demographic changes in those who were symptomatic during the SARS-CoV-2 pandemic or a lower testing threshold for older patients.

### After COVID-19 emerged in Alberta, the positivity rates of endemic coronaviruses decreased

**Fig. 3:**
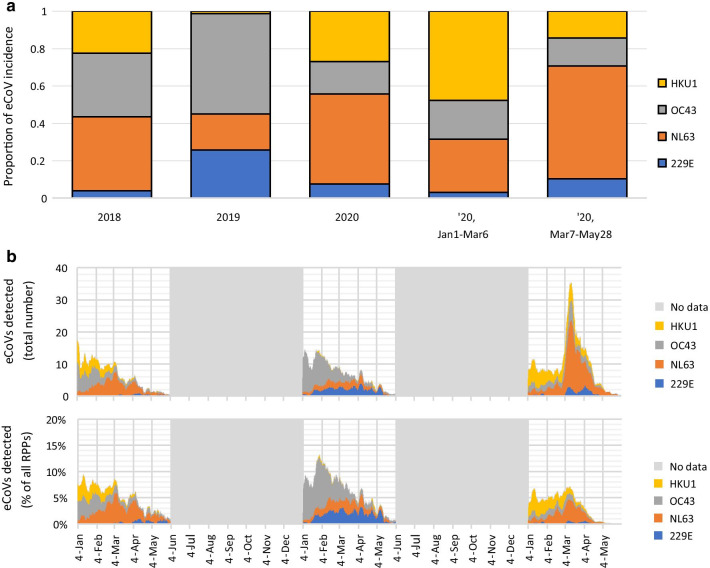
Prevalence of different eCoVs among respiratory specimens in the study period. **a** Predominance of each eCoV in each year 2018–2020. **b** Prevalence of each eCoV over each January–June (2018–2020), number and percent of all RPPs done. *eCoV* endemic coronavirus, *RPP* Respiratory Pathogen Panel

Lastly, the incidence of tests positive for both SARS-CoV-2 and an eCoV was assessed. Between March 7–May 28, 2020, test volumes fluctuated (Fig. 2d). While the proportion of eCoV-positive specimens decreased over this period, SARS-CoV-2 positivity increased without a corresponding increase in specimens positive for both SARS-CoV-2 and an eCoV, simultaneously, suggesting the presence of additional influencing factors such as social factors or competitive exclusion between these coronaviruses (Fig. 2d). Overall, the predominant eCoVs in Alberta during the COVID-19 pandemic were consistent with the two most common eCoVs in Alberta in prior years: NL63 in 2018 and 2020, and OC43 in 2019, with notable variation over the course of each season (Fig. 3a, b).

## Discussion

The COVID-19 pandemic has seen unprecedented levels of respiratory testing, driven by the need to identify cases to control further spread. In the three months after COVID-19 was first reported in Alberta, Canada, nearly 300,000 respiratory specimens were tested for SARS-CoV-2. In parallel, the provincial public health laboratory maintained broad, syndromic nucleic acid testing for additional viral and bacterial pathogens, rapidly amassing data on 18 respiratory pathogens from 52,285 respiratory specimens, including SARS-CoV-2. As a result, to our knowledge this study represents the largest single-year eCoV study [2–5] and by far the largest eCoV study during the COVID-19 pandemic [7–13]. This broad testing approach helps to address a pivotal diagnostic gap amidst the emergence of a novel pathogen: co-infection and possible cross-reactivity with other pathogens that can cause similar clinical presentations. Using a large number of clinical specimens from a real-world setting that may capture true, biological co-infections, this study complements the essential in-lab validations that initially establish test specificity using a small set of known controls. Here, less than 0.01% of specimens tested positive for both SARS-CoV-2 and an eCoV, indicating no significant co-infection or cross-reactivity between SARS-CoV-2 and our four most common eCoVs.

This study identified NL63 as the predominant eCoV in Alberta in both 2018 and 2020, both before and during the COVID-19 pandemic; this is consistent with Albertan findings between 2009 and 2012 [2]. While NL63 was the predominant eCoV (both in specimens where SARS-CoV-2 was and was not identified), other pathogens like enterovirus/rhinovirus were identified more frequently. Altogether, our data supports no cross reactivity between SARS-CoV-2 (which emerged after the RPP manufacturer validation) and other respiratory pathogen targets on the RPP assay, including four other coronaviruses.

By examining concurrent respiratory virus circulation during the COVID-19 pandemic, this study demonstrates the low number of SARS-CoV-2 co-infections in this population and builds upon smaller studies from around the world that have also reported lower viral co-infection rates in SARS-CoV-2-positive vs. SARS-CoV-2-negative patients [7, 10–12]. The results of this study corroborate those of Nowak et al*.*, wherein other respiratory pathogens were detected in < 3% and 13.1% of SARS-CoV-2-positive *vs. -*negative specimens, respectively [10], compared to 3.4% and 10.6% in this study. Notably, the rate of SARS-CoV-2 co-infection with *Mycoplasma pneumoniae* was lower here than reported elsewhere in the literature [6, 13, 24–27]. As such, the role of viral exclusion and cross-protection may be interesting topics for future research [28]. Overall, in contrast to the diagnostic, operational, and surveillance benefits of multiplex syndromic testing in non-pandemic years, broad panels had limited clinical or surveillance value in this pandemic setting.

Importantly, this study illustrates the challenges in performing direct, year-to-year comparisons of broad surveillance data for respiratory viruses. The emergence of the COVID-19 pandemic and the ensuing public health interventions dramatically changed how populations interact, travel, and work, thereby impacting viral spread, the underlying demographics of those requiring testing, and testing volumes. Testing volumes in 2020 substantially increased the underlying statistical power, confounding year-to-year surveillance. For instance, although the eCoV positivity rate among respiratory specimens in 2020 was comparable with prior studies [2, 29], the statistically significant differences in the demographics, location, and age of patients meeting the RPP testing criteria in 2020 compared with previous years was a natural limitation to this study. This highlights a challenge in continuing broad-range (i.e. non-COVID-19) surveillance through this or future pandemics.

The unprecedented testing volumes in Alberta have also highlighted the substantial cost and inflexibility of sustaining fixed, broad-range testing approaches used in prior (non-pandemic) years. More flexible test panel designs and algorithms could better capture local epidemiology and changing needs, whether that is to identify circulating pathogens in routine ‘surveillance mode’ or with panels focused on public health, infection prevention and control, and novel pathogens in ‘pandemic mode.’ Ultimately, more flexible panel designs may support clinical and operational effectiveness in diagnostic laboratories, both during ‘surveillance mode’ (when panels are appropriately informed and customized by periodic local audits), and critically in ‘pandemic mode’ (including when novel pathogens like SARS-CoV-2 emerge). Indeed, flexible syndromic panels that identify pathogens of public health and infection control concern (e.g. SARS-CoV-2, influenza viruses) would be more easily adaptable to novel pathogens and could play an important role in the current pandemic response and future pandemic preparedness.

During a pandemic, clinical laboratories require greater test capacity to support public health efforts to limit further spread [14, 30]. With ongoing surges in COVID-19 cases, laboratories must focus finite resources on targeted pathogens with direct consequences on patient treatment or infection prevention and control.

This study highlights the importance of ongoing diagnostic stewardship to best align laboratory resources with public health efforts. Consequently, as of May 29, 2020, our laboratory pandemic response shifted from routine ‘surveillance mode’ to a prioritized ‘pandemic mode’ by no longer routinely performing the RPP multiplex test with every SARS-CoV-2 test. This further demonstrates how clinical laboratories have adapted throughout this pandemic, as laboratory-driven alternatives continue to mitigate the myriad of ongoing challenges such as test supply shortages [16, 31, 32]. As test volumes increase with the upcoming influenza season, diagnostic stewardship is a key strategy to prioritize public health as the pandemic continues.

## Conclusions

By maintaining broad respiratory pathogen testing from previous years through the emergence of SARS-CoV-2, this study reveals concurrent respiratory virus circulation during the COVID-19 pandemic. Due to the massive surge in test volumes, this became the largest single-year eCoV study and the largest eCoV study during the COVID-19 pandemic. There was no evidence of assay cross-reactivity in a clinical setting between SARS-CoV-2 and the 17 respiratory pathogen targets, and less than 0.01% of specimens tested positive for both SARS-CoV-2 and any eCoV. In fact, specimens containing SARS-CoV-2 had a significantly lower viral co-infections rate. Overall, broad panels had limited clinical or surveillance value and high cost in this pandemic, and more flexible panel designs may better support clinical and operational effectiveness in diagnostic laboratories.

As a consequence of this data, our provincial laboratory eliminated reflexive multiplex testing in a shift from routine ‘surveillance mode’ to a prioritized ‘pandemic mode.’ Focused diagnostic stewardship can help mitigate ongoing challenges to better address future case surges—including future waves of COVID-19—without compromising public health benefits of ongoing testing.

## Data Availability

The datasets used in the current study are available from the corresponding author on reasonable request.

## Abbreviations

COVID-19: Coronavirus-associated disease 2019
eCoV: Endemic coronavirus
LTC: Long-term care
NAT: Nucleic acid test
RPP: Luminex Respiratory Pathogen Panel
RSV: Respiratory syncytial virus
PIV: Parainfluenza virus
ProvLab: Provincial Public Health Laboratory
SARS-CoV-2: Severe acute respiratory syndrome coronavirus 2
Sn: Sensitivity

## Footnotes

1 A retrospective study was performed after this manuscript was initially prepared, in which specimens submitted for respiratory virus testing from December 1, 2019–March 7, 2020 were tested for potentially missed SARS-CoV-2 infections prior to the first routinely-reported case. The study identified a single previously undetected case on February 24, 2020 [23], suggesting that the current study period accurately captures the emergence of COVID-19 in Alberta, Canada.
